# Supplementary material for: Correlation between Antimicrobial Activity Values and Total Phenolic Content/Antioxidant Activity in Rubus idaeus L
Source: Plants (Basel). 2024 Feb 11;13(4):504. doi: 10.3390/plants13040504 (PMC10891700; doi:10.3390/plants13040504)
Supplement: Supplementary file 1 [file plants-13-00504-s001.zip › plants-2866114-supplementary.pdf]

SUPPLEMENT 1

Scheme I. Antioxidant activity (DPPH, ABTS, FRAP), total phenolic content (TPC) and HPLC analysis process and methods

DPPH

- The DPPH$^{\bullet}$ (2,2-Diphenyl-1-picrylhydrazyl) free radical scavenging activity was established using the method suggested by Brand Williams, Cuvelie, and Berset [30], with some modifications: 2 mL DPPH$^{\bullet}$· solution in 96.0% v/v ethanol was mixed with 20 µL of samples. A decrease in absorbance was measured at 515 nm using a Cintra 202 (GBC Scientific Equipment, Knox, Australia) spectrophotometer after 30 min.

ABTS

- An ABTS$^{\bullet+}$ (2,20-azino-bis (3-ethylbenzthiazoline-6-sulphonic acid)) radical cation decolorization assay was adjusted according to the methodology described by Re and colleagues, with some modifications. A volume of 3 mL of ABTS$^{\bullet+}$) solution (absorbance 0.800 ± 0.02) was mixed with 20 µL of samples. The absorbance decrease of each sample was measured at 734 nm in a Cintra 202 (GBC Scientific Equipment, Knox, Braeside, VIC, Australia) spectrophotometer after 30 min.

FRAP

- The ferric-reducing antioxidant power (FRAP) assay was accomplished as described by Benzie and Strain, with some modifications. The FRAP solution consisted of TPTZ (0.01 M dissolved in 0.04 M HCl), $FeCl_3 \times 6H_2O$ (0.02 M in water), and acetate buffer (0.3 M, pH 3.6) at the ratio of 1:1:10. A volume of 3 mL of a recently prepared FRAP reagent was mixed with 2 µL of samples. The absorbance increase was established at 593 nm in a Cintra 202 (GBC Scientific Equipment, Knox, Australia) spectrophotometer after 30 min. Calculation of all antioxidant activity assays was carried out using Trolox calibration curves, and expressed as µmol of the Trolox equivalent (TE) per one gram of dry weight (µmol TE/g DW).

TPC

- The total phenolic content (TPC) of the raspberry extracts was obtained using the Folin–Ciocalteu method, as previously reported by Bobinaite et al. [31]. In short, the test tubes were filled with 1.0 mL of appropriately diluted extract and mixed with 5.0 mL of Folin–Ciocalteu's phenol reagent diluted in distilled water (1/10, v/v) and 4.0 mL of Na_2CO_3 (7.5%). The test solution was left to incubate for 60 min, protected from light exposure, and measured at 765 nm with a Genesys-10 UV/Vis spectrophotometer (Thermo Spectronic, Rochester, NY, USA). The results were presented in mg of gallic acid equivalents (GAE) per 100 g of berries fresh weight (FW); to calculate the results, we used gallic acid as the standard for the calibration curve.

HPLC-PDA

- HPLC analysis was performed using a "Waters e2695 Alliance system" (Waters, Milford, MA, USA) with a photodiode array detector "Waters 2998" according to the HPLC–PDA method for phenolic compounds reported by Raudone et al. [32]. Briefly, the"ACE"(ACT, UK) column (C18,150 mm × 4.6 mm, particle size 3 µm) column was used. The gradient consisted of eluent A (0.05% trifluoracetic acid) and B (acetonitrile) and followed: 0–5 min–12% B, 5–50 min–12–30% B, 50–51 min–30–90% B, 51–56 min–90% B, and 57 min–12% B with the flow rate–0.5 mL/min and injection volume–10µL. The analyte and reference compound retention time and UV absorption spectra were used for peak identification.
